# Microstructural Variation of Clay during Land Subsidence and the Correlation between Macroscopic and Microscopic Parameters

**DOI:** 10.3390/ma15051817

**Published:** 2022-02-28

**Authors:** Shengtong Di, Chao Jia, Pengpeng Ding, Xiao Zhu

**Affiliations:** Institute of Marine Science and Technology, Shandong University, Qingdao 266237, China; pengpengd@163.com (P.D.); 17615209995@163.com (X.Z.)

**Keywords:** land subsidence, scanning electron microscope, microstructural variation, different magnifications, macro–micro parameters

## Abstract

The nonlinear deformation, visco-elasto-plasticity and other macroscopic properties of soil are the concentrated manifestations of its microstructural state. In order to study the microstructural characteristics and variations of the clay under the action of additional stress caused by groundwater exploitation, borehole sampling was carried out on the clay layers at different depths in a typical land subsidence area. Consolidation tests, freeze-drying, ion sputtering, and scanning electron microscope (SEM) were conducted in order to scan and analyze the microstructure of the test samples at different scales. The Particles and Cracks Analysis System (PCAS) was used to quantify the microscopic parameters, the variations of the microstructural parameters with consolidation loads at different sizes were revealed, and the correlation between the macroscopic and microscopic parameters were discussed. The results show that: (1) the microstructural characteristics of soils with different buried depths have directivity, to a certain extent; (2) as the consolidation load increases, the average unit area and average form factor of the soil microstructure generally decrease, the structural arrangement of the unit gradually tends to be orderly, and the average pore area, apparent void ratio and the number of pores generally show a decreasing trend; (3) under the action of a consolidation load, when the microstructure at a relatively large scale is basically stable, the microstructure at a smaller scale will continue to adjust; (4) the apparent void ratio has a good linear regression relationship with the conventional void ratio, and the apparent void ratio has a good exponential growth relationship with the compressibility.

## 1. Introduction

Land subsidence refers to a kind of slowly-changing geological disaster caused by the consolidation and compression of the underground loose rock and soil layer under the action of natural factors or human activities [1,2], which leads to the reduction of the ground elevation in a certain area. It has the characteristics of a large influence range, long duration, slow development and complicated causes. The excessive exploitation of groundwater resources is the main cause of land subsidence [3,4,5,6]. When Tolman and Poland studied land subsidence in the Santa Clara Valley, they found that the compression of the clay layer is an important factor in land subsidence, and its recoverability mainly depends on the structure and mineral composition of the clay layer [7]. The Venice drilling in 1970 revealed that the thickness of the impermeable layer accounted for 51% of the total thickness, but its subsidence value accounted for 84% of the total subsidence value, indicating that the deformation of the impermeable layer played an important role [8]. In the analysis of the soil layer structure in the depth range of 300–800 m in the Lubei Plain, Liu Yong believed that the deformation of sandy soil accounted for only 10%–15% of the total subsistence value, and the land subsidence caused by groundwater exploitation mainly occurred in the clay layer [9].

The main contribution layer of land subsidence determines the adjustment and optimization of the groundwater exploitation plan and the land subsidence prevention scheme [10]. Its deformation and subsidence characteristics—such as nonlinear and visco-elastic–plastic characteristics—are the concentrated manifestation of the adjustment of the soil microstructure [11,12]. The structure of the soil is determined by the shape, size, arrangement and contact mode of the particles and pores in the soil [13]. The different mineral components, stress history and other factors in each region lead to the different internal microstructures of the soil, which directly affects characteristics such as its seepage, consolidation and deformation [14,15].

Some tools for the quantitative analysis of the soil microstructure based on digital image processing techniques have been developed and applied to the geometric and morphological analysis of the soil microstructure [16,17,18]. Test methods such as X-ray diffraction, double oedometer tests, and scanning electron microscopy (SEM) were combined to analyze the evolution of the microstructure of loess during collapse, in order to explain the mechanism of loess collapse [19,20,21]. Methods such as mercury intrusion porosimetry (MIP) and SEM were used to study the deformation and microstructural mechanism of soft soil under cyclic loading, and the variation of the microstructural parameters in the deformation process of soft soil was discussed [22,23]. Conventional tests such as unconsolidated–undrained and direct shear tests, as well as the SEM method, were used to analyze the correlation between the shear characteristics of red clay and the change laws of the microstructure from qualitative and quantitative perspectives [24,25].

The mechanical properties of soil are closely related to its microstructure. The relationship between the shear characteristics of soil and its microstructural changes was discussed by some scholars [26,27]. Moreover, the dynamic characteristics of loess and the variation of the characteristics in the micro-mechanisms therein have also been explored [28,29]. The shrinkage characteristics of soil also affected the soil microstructure when measuring the SWCC (soil–water characteristic curve) by the centrifugal method [30].

Some scholars have also studied the microstructure of soil under different engineering conditions. By introducing the plane fractal dimension, the relationship between the microstructural parameters and the macroscopic deformation of the silty soil around a tunnel under subway loading was established [31]. The micro-characteristics and meso-shear mechanism of the soils of a slip zone in a landslide were studied with an X-Ray diffractometer and a scanning electron microscope [32]. The pore water pressure and microstructures of silty clay under freeze–thaw cycles were tested [33].

The hysteresis effect of land subsidence is closely related to the creep properties of clay [34]. It is very important to study the essential mechanism of clay consolidation and deformation under the action of the additional stress caused by groundwater exploitation, and to reveal the evolution characteristics and rules of the microstructure of soil deformation and settlement during the occurrence and development of land subsidence. Therefore, borehole sampling was carried out in Dezhou, an important affected area of land subsidence in the North China Plain. Then, conventional physical mechanics tests such as consolidation and compression were performed, and the freeze-drying method and ion sputtering method were used to prepare test samples. The environmental electron microscope scanning technique was used to analyze the microstructure of the clay under different consolidation loads. Finally, the parameters of the microstructure were quantitatively extracted and analyzed by adopting the PCAS, the variations of the soil microstructure under different consolidation loads were revealed, and the variation characteristics of the soil microstructure under different sizes were explored and analyzed. Compared with the experimental results of conventional consolidation compression, the correlation between the microstructural parameters and the macroscopic parameters was analyzed.

## 2. Materials and Methods

Borehole sampling was carried out at the new base of Shandong Dezhou Lubei Geological Engineering Survey Institute (Shandong Deshui Geothermal Technology Co., Ltd., Dezhou, China), a typical representative area of land subsidence in the North China Plain, and clay layers with different buried depths were selected as the research objects. The conventional physical parameters such as the density, porosity, compressibility and permeability coefficient, and conventional mechanical parameters such as the cohesion and internal friction angle were tested. On this basis, the scanning electron microscopy experiments on the undisturbed soil and the soil under different consolidation loads were carried out. During the scanning electron microscope experiments, different magnifications were used to study the variations of the microstructure of the soil at different scales.

**Figure 1:**
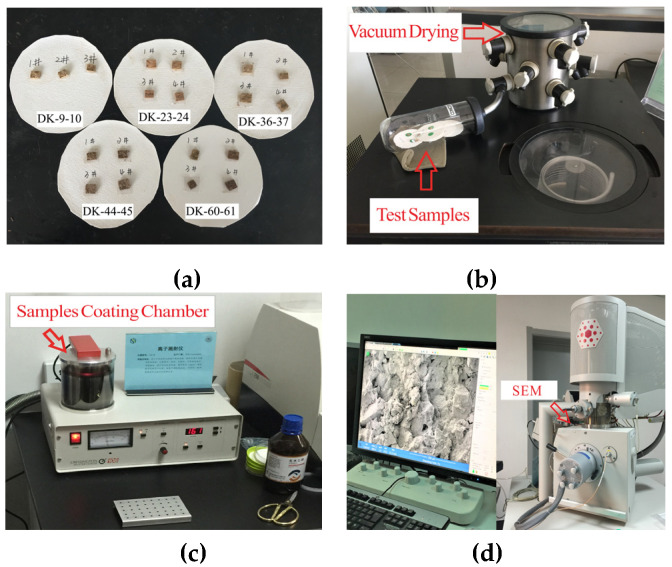
The main test procedures of the microstructure scanning samples. (**a**) Sample preparation; (**b**) freeze-drying; (**c**) surface coating; (**d**) scanning electron microscopy.

The sample preparation and microstructure scanning of undisturbed clay and compressed clay with consolidation loads of 100 kPa, 400 kPa, 800 kPa and 1600 kPa were carried out by means of a freezer-dryer (Shandong University, Jinan, China), an ion sputtering apparatus (Shandong University, Jinan, China) and a SEM micro-electron microscope scanner (Shandong University, Jinan, China). The main test procedures included sample preparation, freeze-drying, surface coating, and scanning electron microscopy, as shown in Figure 1. The freeze-drying method was intended to sublimate and extract the pore water in the soil in a low-temperature environment and a vacuum, so as to avoid the damage of the original soil microstructure by water evaporation in the drying process. The electron gun emits electrons to the surface of the sample during the test of the electron microscope scanner, and the electrons need to collide with the atoms on the sample surface in order to generate electronic signals, which are then processed by the detection system to form the surface information of the sample. Therefore, it was necessary to conduct sputtering coating on the surface of the test samples to make their surfaces conductive.

According to Shear’s classification of clay microscopic pores, the pores with a pore size less than 0.014 μm were considered to be intragranular pores, the pores with a pore size between 0.014 μm and 1.8 μm were considered to be intergranular pores, the pores with pore size between 1.8 μm and 70 μm were considered to be intra-aggregate pores, and the pores with pore size between 70 μm and 4000 μm were considered to be inter-aggregate pores [35]. Combined with the sizes of the samples, four magnifications of 800, 2000, 10,000, and 20,000 times were set during the SEM experiments in this study, which were used to observe and analyze the distribution characteristics between the particles and aggregates. The microstructures of the undisturbed soil were observed and analyzed from the vertical and horizontal directions, and the microstructures of the compressed soil samples were observed and analyzed from the vertical direction. Test soil samples of different groups are numbered, and the specific experimental schemes are shown in Table 1.

## 3. Conventional Physical and Mechanical Characteristics

Conventional physical and mechanical experiments were carried out on the parameters of five groups of test samples, measuring their gravity, porosity, compressibility, permeability coefficient, cohesion, and internal friction angle. The results are shown in Table 2.

It can be seen from Table 2 that the natural density of the five groups of soils at different depths is basically the same, at about 21 kN/m^3^, and the porosity has no obvious regularity, with an average of about 35.7%. The compressibility generally decreases with the buried depth, indicating that the compressibility of the soil becomes lower and lower as the depth increases, and the permeability coefficient is basically below 10^−6^ cm/s. The preconsolidation pressure shows an increasing trend with the increase of depth, and it was judged to be under-consolidated soil based on the corresponding self-weight stress. In addition, the cohesion gradually increases with the increase of the buried depth, and the internal friction angle gradually decreases with the increase of the buried depth. In general, with the increase of the buried depth, the compressibility, permeability coefficient and internal friction angle of the five groups of soil samples generally show a decreasing trend, while the preconsolidation pressure and cohesion generally show an increasing trend, and the natural density and porosity have no obvious change rules.

**Figure 2:**
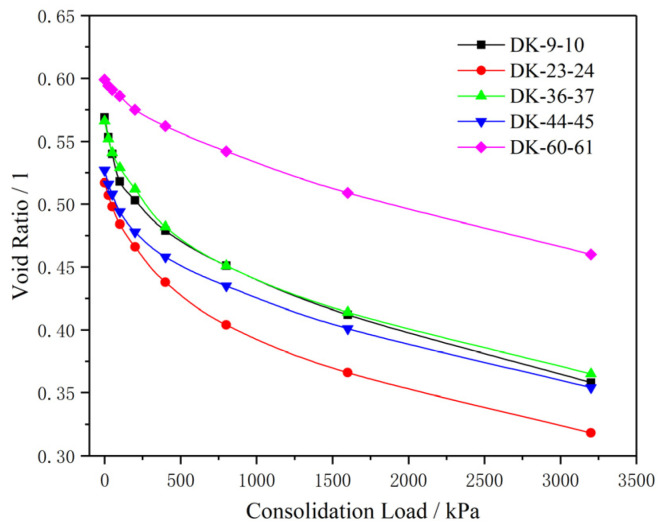
The e–p consolidation compression curves of the samples.

Figure 2 shows the variation of the clay void ratio with the consolidation load. It can be seen from the figure that the void ratio of the clay body shows a decreasing trend with the consolidation load, and the decreasing rate gradually slows down. The average compression moduli of the five groups of samples with the consolidation load between 100 kPa and 200 kPa are 10.46 MPa, 8.43 MPa, 9.21 MPa, 9.55 MPa and 14.54 MPa, respectively, and the average compression moduli of the samples with the consolidation load between 200 kPa and 400 kPa are 13.08 MPa, 10.84 MPa, 10.44 MPa, 15.27 MPa and 24.6 MPa, respectively. The average compression moduli of the samples with the consolidation load between 400 kPa and 800 kPa are 22.41 MPa, 17.85 MPa, 20.21 MPa, 26.56 MPa and 31.98 MPa, respectively, and the average compression moduli of the samples with the consolidation load between 800 kPa and 1600 kPa are 32.18 MPa, 31.94 MPa, 33.86 MPa, 35.93 MPa and 38.76 MPa, respectively. It can be seen that the compression modulus of the soil increases step by step with the increase of the consolidation load, and the increasing trend accelerates step by step. Under the same consolidation load, the compression modulus generally increases with the increase of the buried depth.

## 4. Microstructural Characteristics

The concept of the microstructure of cohesive soil was first used by Terzaghi in 1925, and Kubiena gave a precise definition of a microstructure in 1938, which was called “fabric”. Later, Brewer proposed the concept of a fabric or structure in 1964 [36], and defined it as the physical composition of the soil shown by the basic particles that form composite particles and the corresponding pore size and shape arrangement, which further improved the definition of the concept of a soil microstructure. At present, the research on microstructural features mainly include four aspects: structural unit features, particle arrangement features, pore features, and structural connection features.

**Figure 3:**
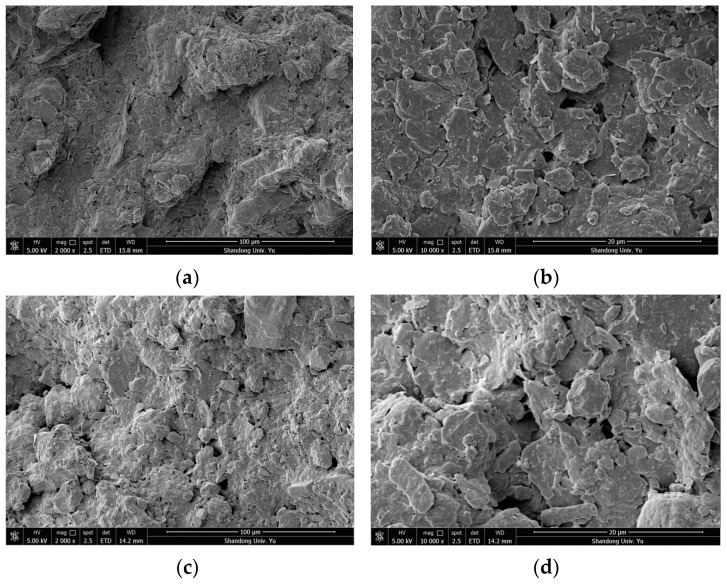
SEM images of the representative microstructure of the DK-9-10 group. (**a**) 2000 times, in the vertical direction; (**b**) 10,000 times, in the vertical direction; (**c**) 2000 times, in the horizontal direction; (**d**) 10,000 times, in the horizontal direction.

**Figure 4:**
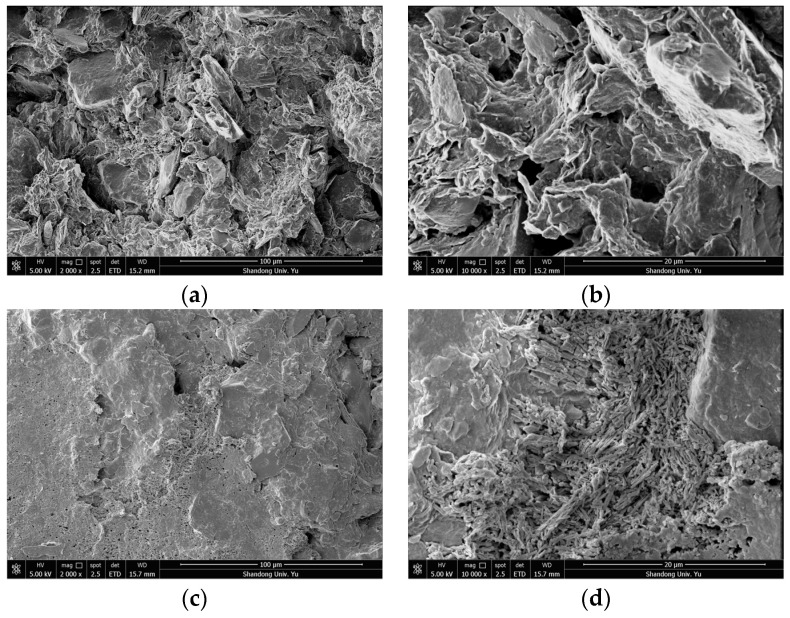
SEM images of the representative microstructure of the DK-44-45 group. (**a**) 2000 times, in the vertical direction; (**b**) 10,000 times, in the vertical direction; (**c**) 2000 times, in the horizontal direction; (**d**) 10,000 times, in the horizontal direction.

In order to study the microstructural characteristics of the clay bodies with different buried depths, and to reveal the characteristics of the clay in this area—such as the contact and arrangement of particles (aggregates), pore characteristics and structure distribution—the microstructural characteristics of undisturbed soil samples on the vertical and horizontal planes were analyzed in this section. Combined with the sample sizes (length × width = 8 mm × 6 mm) and Shear’s criteria for the classification of clay microscopic pores, scanned images with a magnification of 2000 times and a magnification of 10,000 times were selected in order to analyze the intergranular microstructural characteristics and intra-aggregate microstructural characteristics of the clay. Figure 3 shows the SEM images of the representative microstructure of the group DK-9-10 when magnified 2000 and 10,000 times in the vertical and horizontal directions, respectively. Likewise, Figure 4 shows the SEM images of the representative microstructure of the group DK-44-45 when magnified 2000 and 10,000 times in the vertical and horizontal directions, respectively.

It can be seen from the comprehensive analysis that the unit characteristics, pore characteristics and connection characteristics of the microstructure of the soil at different depths were different, indicating that the deposition history or genesis of the soil at different depths was different. The microstructural characteristics and pore characteristics of the structural units at different scales can be observed intuitively from SEM images with different magnifications, which makes SEM scanning results both representative and universal. By comparing the microstructures of the soils in the horizontal and vertical directions, it was found that the microstructures of soils at different buried depths were different. Among them, the group DK-9-10 with a buried depth of 74.2–74.3 m and the group DK-44-45 with a buried depth of 270.4–270.6 m show more obvious anisotropy, while the other groups show no obvious difference in the vertical and horizontal directions.

## 5. Microstructural Parameters

In this section, the microstructural characteristics of the soil are quantified, and the representative microstructural parameters are selected to carry out the quantitative analysis of the microstructure of the images with SEM magnifications of 2000 times and 10,000 times. The correlation between different consolidation loads and soil microstructural parameters is analyzed, and the influence of the consolidation loads on the evolution of the soil microstructural units and pores at different scales is studied.

According to the main components of soil microstructural characteristics, combined with the main macroscopic properties of soil, such as its compressibility and permeability, three microstructural parameters—i.e., the average unit area, unit shape coefficient and probability entropy—were selected in order to study the variations of the size, shape and structural arrangement of microstructural units with the changes of the consolidation loads. Meanwhile, three microstructural parameters—the average pore area, pore number and apparent pore ratio—were selected to study the evolution of the pore size, pore content and porosity of the soil microstructure under different consolidation loads.

### 5.1. Definition of the Microstructural Parameters

The average unit area $\bar{A_{u}}$ is the average area of the unit skeleton of the soil microstructure scanned in the SEM picture, and the calculation formula is as follows:

(1) $$\bar{A_{u}}=\frac{1}{n}\sum_{i=1}^{n}A_{i}^{u}$$

where *n* is the number of microstructural units, and $A_{i}^{u}$ is the area of the i-th structural unit.

The unit shape coefficient $F_{f}$ is defined as the ratio of the circumference of a circle with the same area as the unit to the actual circumference of the unit. Here, the average shape coefficient, $\bar{F_{f}}$, of the unit body with statistical average significance is also used to describe the shape of the soil structure unit body. The calculation formula is as follows:

(2) $$\bar{F_{f}}=\frac{1}{n}\sum_{i=1}^{n}\frac{C_{i}}{S_{i}}$$

Here, *n* is the number of microstructural units, $C_{i}$ is the circumference of a circle with the same area as the i-th structural unit, and $S_{i}$ is the actual circumference of the i-th structural unit. It can be seen that the value range of $\bar{F_{f}}$ is between 0 and 1. The smaller the value, the more oblate the unit structure, and the larger the value, the closer the unit structure is to a circle.

The directional distribution frequency $F_{d}^{i}$ of the unit is used to describe the distribution intensity of the unit in the i-th location direction. The angle between the long axis direction of the unit body and the horizontal direction is the directional angle of the unit body, and the value range of the direction angle is [0;180]. It can be divided into n locations, and each location represents a directional angle range of 180/*n*. Then, the directional frequency of the units distributed in each location can be obtained by statistics; the calculation formula is

(3) $$F_{d}^{i}=m/M*100\%$$

Here, *m* is the number of units with the directional angle distributed at the i-th location, and *M* is the total number of sample units.

The probability entropy $E_{p}$ is the quantitative integration of the directional angle of the unit body and the directional distribution frequency, $F_{d}^{i}$, which is used to quantitatively describe the overall directionality of the unit body or pore system, and is defined as

(4) $$E_{p}=-\sum_{i=1}^{n}F_{d}^{i}\log_{n}F_{d}^{i}$$

wherein, $F_{d}^{i}$ is the distribution frequency of the unit in the i-th location direction, and *n* is the number of divided locations. The value domain of the probability entropy $E_{p}$ is between 0 and 1. When $E_{p}$ is 0, it indicates that all of the units are in the same direction. When $E_{p}$ is 1, it indicates that all of the units are uniformly distributed in all directions. The larger the $E_{p}$, the more disordered and random the structure, and the worse the directionality.

The average pore area $\bar{A_{v}}$ is similar to the average unit area $\bar{A_{u}}$, which is the average area of all of the pores in the soil microstructure scanned in the SEM picture. It is a quantitative index with statistical significance to describe the average size of the pores in the microstructure of the soil, and its calculation formula is

(5) $$\bar{A_{v}}=\frac{1}{n}\sum_{i=1}^{n}A_{i}^{v}$$

wherein *n* is the number of pores, and $A_{i}^{v}$ is the area of the i-th pore.

The apparent void ratio $V_{a}$ is used to describe the porosity of the soil microstructure in the SEM scan image. It is defined as the ratio of the area occupied by the pores to the area occupied by the units in the SEM scan image; its calculation formula is

(6) $$V_{a}=\frac{S_{v}}{S_{s}}$$

wherein $S_{v}$ is the area occupied by pores in the SEM image, and $S_{s}$ is the area occupied by units in the SEM image.

### 5.2. Methods and Steps of the Parameter Quantification

The PCAS image recognition and analysis system developed by the team of Chun Liu from Nanjing University was used to extract the microstructural parameters of the clay body. The parameter extraction process mainly included three steps: image preprocessing, threshold segmentation processing, and morphological processing [37,38]. A relatively small threshold of 60–100 should be selected when the black area is used as a pore for the quantitative study of the pore structure, while a relatively large threshold should be selected when the soil particle morphology is studied, and the recommended range is 150–220 [39]. In the process of image processing using GIS to study the microstructure of cohesive soil, Wang Baojun also found that the larger the threshold value, the larger the pore area, and the smaller the threshold value, and the larger the particle size [40].

**Figure 5:**
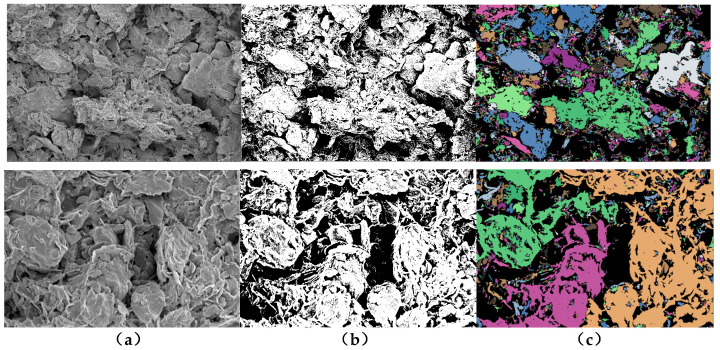
The process of the extraction and analysis of the microstructural parameters (the above is magnified 2000 times, and 10,000 times below). (**a**) Image preprocessing; (**b**) threshold segmentation; (**c**) morphological processing.

In this paper, the black areas are treated as pores during the quantitative analysis of the SEM microstructure. When processing the pore structure, a relatively small threshold can be used to better distinguish the unit bodies and pores in the microstructure, in order to obtain more accurate pore characteristics. When analyzing the morphology and structural arrangement of the microstructural units, a relatively large threshold can be used to better distinguish the shape, size and arrangement characteristics of the units. Therefore, different thresholds were adopted for quantitative analysis of the microstructure of particles and pores in order to obtain more realistic results. The analysis steps in the process of the microstructural parameter extraction are shown in Figure 5.

### 5.3. Variations of the Microstructural Parameters

#### 5.3.1. Average Unit Area

**Figure 6:**
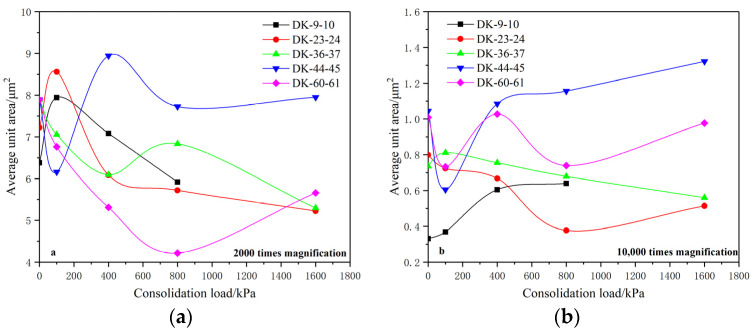
Relationship between the average unit area and the consolidation load. (**a**) 2000 times magnification; (**b**) 10,000 times magnification.

Figure 6 shows the variation of the average unit area under different consolidation loads when the soil microstructure is magnified 2000 times and 10,000 times. 

It was found that the average unit area of the five groups of undisturbed soil is between 6–8 μm^2^ when magnified 2000 times. Under the action of the consolidation load, the average unit area generally shows a downward trend, which is manifested by the gradual crushing of the soil particles. For the SEM microstructure under 10,000-times magnification, the average unit area of undisturbed soil is mainly distributed between 0.3–1.1 μm^2^, and the variation laws of the average unit area under a consolidation load are not completely consistent with those under 2000-times magnification.

It can be seen from the above variations of the average unit area that the average unit area magnified 10,000 times is smaller than that at 2000 times, the number of units is relatively less, and the changing trend is more complicated. This is because the different distribution and arrangement of structural units at different scales have different response degrees to external loads, resulting in different adjustment processes such as the compression–fragmentation and compaction–aggregation of the unit under the action of the consolidation load, which leads to the different variations of the unit area with the change of the consolidation loads.

#### 5.3.2. Unit Shape Coefficient

**Figure 7:**
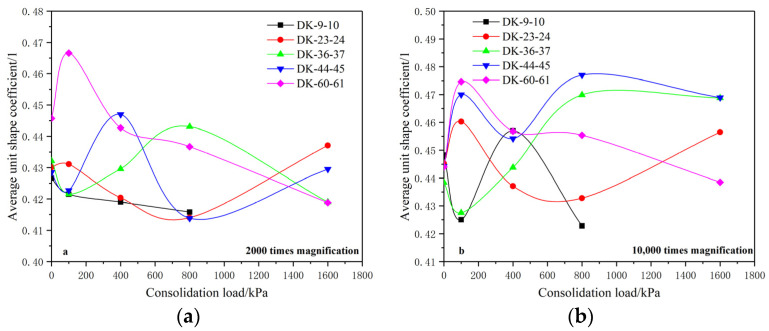
Relationship between the average unit shape coefficient and the consolidation load. (**a**) 2000 times magnification; (**b**) 10,000 times magnification.

The unit shape coefficients of the soil microstructure at magnifications of 2000 and 10,000 times were extracted in order to analyze the influence of a consolidation load on the shape of the soil microstructural unit at different scales, as shown in Figure 7.

The analysis shows that the average unit shape coefficient of the undisturbed soil is basically distributed between 0.425 and 0.45 when the magnification is 2000 times, and the average unit shape coefficient generally shows a downward trend with the increase of the consolidation load. Here, the average unit shape coefficients of group DK-9-10 and group DK-60-61 show a relatively obvious downward trend with the increase of the consolidation load, indicating that the structural units are gradually compressed and structurally adjusted under the action of the consolidation load, gradually becoming more elongated. The average unit shape coefficients of group DK-23-24, group DK-36-37 and group DK-44-45 show a fluctuation phenomenon of decreasing first, then increasing, and then decreasing. This shows that the structural units are compressed and structurally adjusted under the initial consolidation load, which causes the shape to become oblate. Then, as the consolidation load continues to increase, recombination and aggregation occur among the structural units, resulting in the fluctuation of the unit shape coefficients.

For the soil microstructure with a magnification of 10,000 times, the structural unit shape coefficients of the undisturbed soil are basically distributed at about 0.445, and they show obvious fluctuations with the increase of the consolidation load. Among them, group DK-23-24, group DK-36-37, and group DK-44-45 have larger unit shape coefficients when the consolidation load is 1600 kPa than that of the undisturbed soil. The analysis suggests that the shape of the microstructural units is wider and more round when magnified 10,000 times, the number of units is relatively less, the structure distribution is more discrete, and the response degree to different consolidation loads is different. Compression deformation and structural adjustment will cause the shape of the units to become slenderer and the shape coefficient to decrease, while the decomposition and aggregation of the units under the action of a consolidation load may cause the shape of the units to become wider and rounder, and the shape coefficient to increase. Comparing the change laws of the shape coefficients of the microstructural units under magnifications of 2000 times and 10,000 times, it of was found that the shape of the microstructural units at a smaller scale are more sensitive to the load response.

#### 5.3.3. Probability Entropy

**Figure 8:**
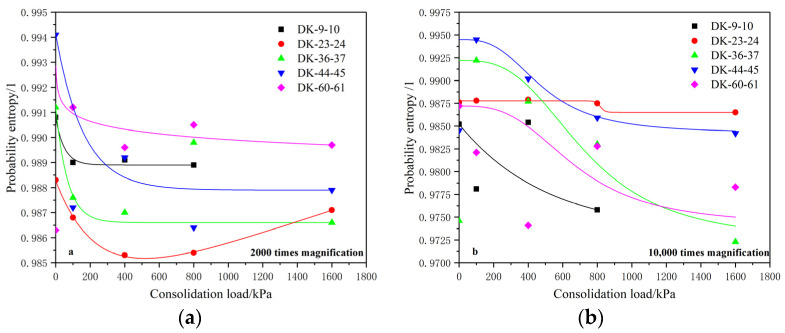
Relationship between the element probability entropy and consolidation load. (**a**) 2000 times magnification; (**b**) 10,000 times magnification.

The probability entropy was extracted in order to study the variations of the structural arrangement of the microstructural units with the consolidation load under different magnifications, as shown in Figure 8. It can be seen from the fitting curve of the probability entropy that under the magnification of 2000 times, the probability entropy of the units shows an obvious decreasing trend with the increase of the consolidation load. The probability entropy decreases rapidly before the consolidation load reaches 400 kPa, and the probability entropy gradually stabilizes after 400 kPa. This shows that before the consolidation load is 400 kPa, the arrangement of the microstructural units is adjusted quickly, and the arrangement remains basically stable after the consolidation load becomes 400 kPa.

Under a magnification of 10,000 times, it can be seen that the probability entropy also shows a significant downward trend with the increase of consolidation load, indicating that the arrangement of units at this scale also tends to be orderly and regular under the action of a consolidation load. The comparative analysis shows that the decrease laws of the probability entropy of units when they are magnified 10,000 times is different from those when they are magnified 2000 times. Under the condition of a magnification of 10,000 times, the probability entropy of the units continues to decrease as the consolidation load increases from 0 to 1600 kPa, and although the decreasing rate decreases, it basically does not reach a stable state.

The analysis shows that the structural units with an average area of 6–8 μm^2^ are arranged and adjusted rapidly when the consolidation load is less than 400 kPa, and the structural adjustment tends to be stable when the consolidation load is greater than 400 kPa. The structural arrangement of the units with an average area of about 0.3–1.1 μm^2^ is continuously adjusted between the consolidation loads of 0 and 1600 kPa, and the adjustment rate tends to decrease gradually. This shows that with the increase of the consolidation load, when the arrangement of the units at a relatively larger scale is basically stable, the arrangement of the units at a smaller scale will continue to be adjusted, indicating that structural units at smaller scales have larger load influence intervals and sensitivity, which is consistent with the analysis results of the average unit shape coefficient.

#### 5.3.4. Average Pore Area

**Figure 9:**
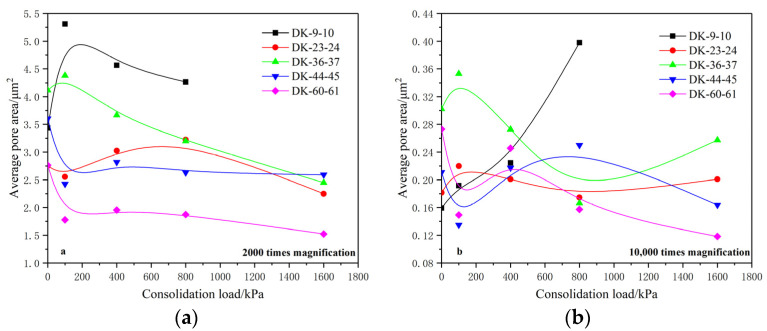
Relationship between the average pore area and consolidation load. (**a**) 2000 times magnification; (**b**) 10,000 times magnification.

The average pore area of the microstructure was extracted in order to study the influence of different consolidation loads on the pore size of the soil. Figure 9 shows the variation of the average pore area of the soil microstructure with a consolidation load under magnifications of 2000 times and 10,000 times.

The analysis showed that the average pore area of the undisturbed soil is distributed between 2.5 and 4.5 μm^2^ when magnified 2000 times, and generally shows a downward trend under the action of a consolidation load. Here, the average pore areas of group DK-9-10, group DK-23-24 and group DK-36-37 show a trend of first increasing and then gradually decreasing. It is believed that a large number of micropores are compressed and disappear at the early stage of a consolidation load, and the number of pores decreases rapidly, resulting in the initial increase of the average pore area. Group DK-44-45 and group DK-60-61 show a trend of rapid decrease at first and then gradually slow and stable, which is caused by the fact that—under the action of a consolidation load—a large number of pores are compressed and reduced, and then tend to be stable. 

Under the magnification of 10,000 times, the average pore area of the undisturbed soil is distributed between 0.15 and 0.35 μm^2^, and the variation of each group is not completely consistent under the action of a consolidation load. The average pore area of each group fluctuates with the increase of the consolidation load, but it still shows a decreasing trend on the whole. Here, the average pore area of group DK-9-10 increases gradually with the consolidation load, which is believed to be caused by the substantial reduction of the number of micropores under the effect of the consolidation load and the generation of new pores during the compression process.

#### 5.3.5. Apparent Void Ratio

**Figure 10:**
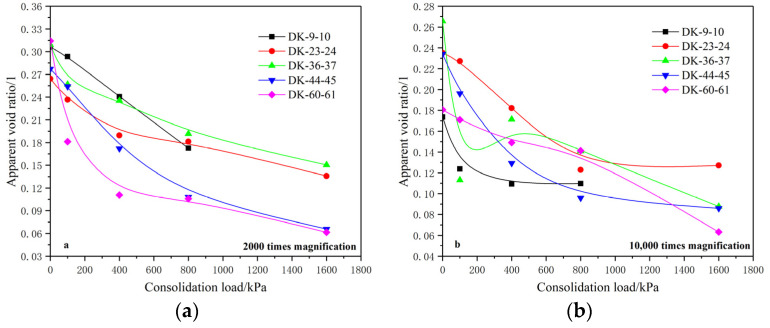
Relationship between the apparent void ratio and the consolidation load. (**a**) 2000 times magnification; (**b**) 10,000 times magnification.

Figure 10 shows the variation of the apparent void ratio of the microstructure with the consolidation load. It was found that the apparent void ratio of the undisturbed soil under a magnification of 2000 times is mainly distributed between 0.25 and 0.32. As the consolidation load increases, the apparent void ratio shows a decreasing “first fast and then slow” trend, and its variation law is close to that of the conventional pore ratio. The apparent void ratio of undisturbed soil is mainly between 0.15 and 0.27 at 10,000-times magnification, and it generally shows a decreasing trend with the increase of the consolidation load, with a few fluctuations, but the general trend is also close to the variation law of the conventional pore ratio.

A comparative analysis of the apparent void ratio and the conventional void ratio of the undisturbed soil under different magnifications shows that the apparent void ratio is between 0.25 and 0.32 when magnified 2000 times, and the apparent void ratio is between 0.15 and 0.27 when magnified 10,000 times. The apparent void ratio at smaller scales is relatively smaller; that is, the ratio of the pore content to the unit content at smaller scales is smaller. Compared with the conventional void ratio of 0.527–0.599, the apparent void ratio is relatively smaller. This is because the apparent pore ratio is the ratio of the pore area to the unit area under the condition of SEM scanning, which has a two-dimensional property. Some of the real three-dimensional pores are shown in the SEM pictures as the undulations of the units, which leads to the apparent void ratio being smaller than the conventional void ratio.

#### 5.3.6. Distribution of the Average Pore Area

In order to analyze specifically the size distribution of the microstructural pores in each group of undisturbed soil and the variations of the size distribution under the action of consolidation, the content distribution of the average pore area under different consolidation loads was statistically analyzed with SEM magnifications of 2000 times and 10,000 times, as shown in Table 3 (2000-times magnification) and Table 4 (10,000-times magnification).

As can be seen from Table 3, the number of pores in each group of undisturbed soil is 1957, 2186, 1649, 1731 and 2481, respectively, when magnified 2000 times, and the number of pores generally decreases with the increase of the consolidation load. The distribution range of the dominant content of the average pore area is 0–2 μm^2^, and the contents of each group in the dominant distribution range are 74.2%, 76.44%, 71.68%, 73.72% and 72.71%, respectively.

As the consolidation load increases, the content of the dominant distribution interval of the average pore area of each group gradually increases, which should include the reduction in the number of pores during compression. The content of pores larger than 8 μm^2^ shows an overall decreasing trend. Taking the consolidation load of 800 kPa as an example, the number of pores larger than 8 μm^2^ in each group is reduced by 38.9%, 21.3%, 40.7%, 55.6% and 80.5% compared with that in the undisturbed soil, respectively. It can be seen that the pores of the soil are generally compressed during the consolidation and compression process. The larger pores are compressed and become smaller, such that the content of larger pores is reduced, and the smaller pores are compacted during the consolidation process, such that the total number of pores gradually decreases.

It can be seen from Table 4 that the number of pores in each group of undisturbed soil is 1064, 1206, 795, 1027, and 643, respectively, when magnified 10,000 times. The distribution range of the dominant content of the average pore area is 0–0.08 μm^2^, and the contents of each group in the dominant distribution range are 70.67%, 71.64%, 67.55%, 67.87% and 62.13%, respectively. With the increase of the consolidation load, the pore content in the dominant interval of each group increases step by step, while the total number of pores decreases gradually. The content of pores larger than 0.32 μm^2^ also shows a decreasing trend. Taking the consolidation load of 800 kPa as an example: the number of pores larger than 0.32 μm^2^ in each group is reduced by 59.7%, 36.5%, 11.8%, 58.0% and 5.6% compared with that in the undisturbed soil, respectively.

It can be seen that the variation of the content of microscopic pores in the soil under a consolidation load when magnified 10,000 times is basically the same as that when magnified 2000 times; that is, the total number of pores and the content of larger pores both show an obvious decreasing trend. This shows that the soil pores are continuously compressed and compacted under the action of the consolidation load, and the content of larger pores is significantly reduced and transformed into small and medium pores. The tiny pores are compacted and disappear during the consolidation process, resulting in a decrease in the total number of pores.

## 6. Correlation between the Macroscopic and Microscopic Parameters

The apparent void ratio, as a microstructural parameter of soil with two-dimensional properties, lacks the spatial distribution characteristics of pores in SEM images compared with the conventional three-dimensional void ratio. The actual three-dimensional pores are reflected in the form of the undulation and roughness of the units in the SEM figure, such that the apparent pore ratio is often smaller than the conventional pore ratio, but the variation law of the two is similar with the increase of the consolidation load. Therefore, two conventional physical test parameters—the conventional void ratio and the compression coefficient—were selected in order to analyze the correlation between the two parameters and the apparent void ratio, and to study the correlation between the microscopic and macroscopic parameters of the soil at different scales under the action of a consolidation load.

**Figure 11:**
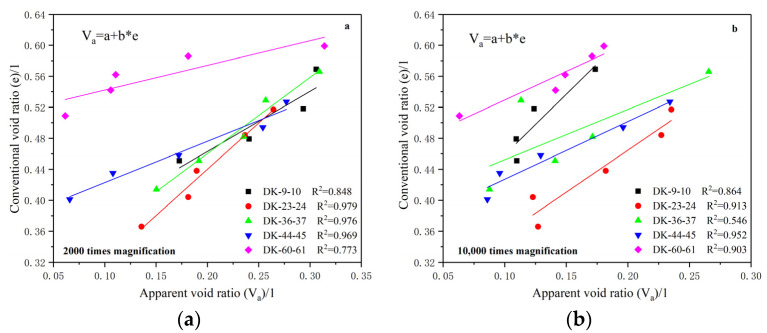
Relationship between the apparent void ratio and the conventional void ratio. (**a**) 2000 times magnification; (**b**) 10,000 times magnification.

Figure 11 shows the fitting relationship curve between the apparent void ratio and the conventional void ratio of each group of soils at different scales. It can be seen that the apparent void ratio has a good linear relationship with the conventional void ratio at the magnification of 2000 times, and the relationship can be described well by the linear equation $V_{a}=a+b*e$, where a and b are fitting constants related to the pore structure characteristics of each soil group. The fitting variance of each group is 0.848, 0.979, 0.976, 0.969 and 0.773, respectively, with an average fitting variance of 0.909. The results show that the variation of the apparent void ratio is basically consistent with that of the conventional void ratio under a consolidation load.

From the correlation between the apparent void ratio of the soil and the conventional void ratio when magnified 10,000 times, it can be seen that the two parameters also basically obey the linear relationship well. The fitting variance of each group is 0.864, 0.913, 0.546, 0.952 and 0.903, respectively, and the average fitting variance is 0.836. The fitting effect is slightly worse than that when magnified 2000 times, which is also related to the larger dispersion and stress response degree of pore characteristics at smaller scales. Comparing the correlation between the apparent pore ratio and the conventional pore ratio under different magnification scales, it can be seen that the relationship between the apparent void ratio and the conventional void ratio when the average pore area is 2.5–4.5 μm^2^ has a better correlation than that when the average pore area is 0.15–0.35 μm^2^. The results indicate that the pores with an average area of 2.5–4.5 μm^2^ may have a more dominant effect on the physical and mechanical properties of the soil than the pores with an average area of 0.15–0.35 μm^2^.

**Figure 12:**
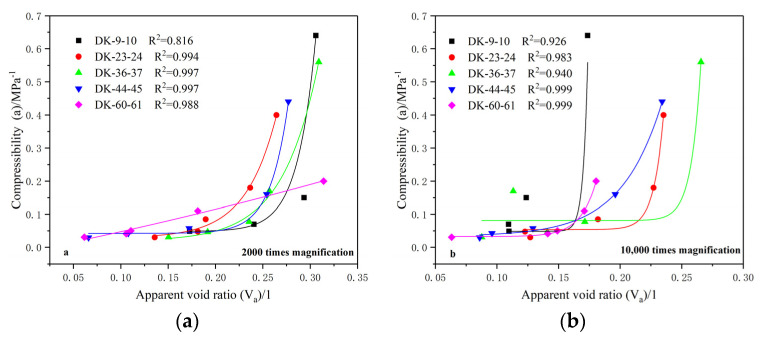
Relationship between the apparent void ratio and compressibility. (**a**) 2000 times magnification; (**b**) 10,000 times magnification.

The change rates of the void ratio between 0–25 kPa, 100–200 kPa, 400–800 kPa, 800–1600 kPa, and 1600–3200 kPa were taken to be the compressibilities of the soil under consolidation loads of 0 kPa, 100 kPa, 400 kPa, 800 kPa and 1600 kPa, respectively. The correlations between the apparent void ratio and the compressibility at different scales were obtained, as shown in Figure 12.

From the correlation between the apparent void ratio and compressibility at a magnification of 2000 times, it can be seen that the apparent void ratio and compressibility exhibit a good exponential growth relationship, and the fitting variances are 0.816, 0.994, 0.997, 0.997 and 0.988, respectively, with an average fitting variance of 0.958. With the increase of the apparent void ratio, the increase rate of the compressibility gradually increases, which is similar to the correlation between the conventional void ratio and the compressibility. The apparent void ratio and compressibility also show an exponential growth relationship at a magnification of 10,000 times, and the fitting variances are 0.926, 0.983, 0.940, 0.999 and 0.999, respectively. The average fitting variance is 0.969, and the fitting effect is also good.

Comparing the correlation between the apparent void ratio and the compressibility at different scales, it can be found that the initial increase in the apparent void ratio has a relatively small effect on the compressibility at a magnification of 10,000 times. When the apparent void ratio increases to a certain value, the compressibility will undergo a rapid increase stage. The results show that a small increase in pores with an average area of 0.15–0.35 μm^2^ has little effect on the overall compressibility of the soil, and only when the void ratio reaches a certain level will it have a significant impact on the compressibility of the soil. In contrast, a small increase in pores with an average area of 2.5–4.5 μm^2^ has a significant impact on the overall compressibility of the soil.

## 7. Discussion

(1) The real structure of soil in nature is usually anisotropic, random and discrete, and whether the scanning electron microscope image selected for the analysis of microstructure parameters is representative or not will affect the accuracy of the analysis results. In addition, the SEM images used for the quantitative analysis of microstructural parameters have two-dimensional properties, and part of the real three-dimensional pores is shown in the SEM pictures as the undulations of the units. Therefore, the selection of the threshold value is a very important factor in the quantitative analysis of the microstructure.
(2) The microstructures of soil under different magnifications are different, and their variation laws with the change of the consolidation load should also be different. In this study, combining the sample size and Shear’s criteria for the classification of clay microscopic pores, the different variation rules of soil microstructural parameters under consolidation load conditions were obtained when the magnification was 2000 times and 10,000 times. The intergranular microstructural characteristics and intra-aggregate micro-structural characteristics were emphatically analyzed. In order to better understand the evolution laws of the microstructure of soil at different sizes, the quantitative analysis of the microstructure under other magnifications is also important.

## 8. Conclusions

(1) The unit characteristics, pore characteristics and microstructural connection characteristics of soils at different depths in the vertical and horizontal directions are not the same, and there is a certain directionality. Several groups of soils with different buried depths have fewer and denser pores in the vertical direction than in the horizontal direction, and some soils have other obvious types of microstructure composition in the horizontal direction.
(2) The average unit area and average shape coefficient of the soil microstructure generally decrease with the increase of the consolidation load, but there are also fluctuations. When the microstructure undergoes a process of polymerization and reconstruction under the action of stress, the average unit area and average shape coefficient will both have a tendency to increase. The microstructure of soil tends to be more orderly with the increase of the consolidation load. The average pore area, apparent void ratio and the number of pores generally show a gradually decreasing trend during the consolidation process.
(3) Under the action of a consolidation load, when the microstructure at a relatively large scale is basically stable, the microstructure at a smaller scale will continue to be adjusted; that is, the microstructural unit at a smaller scale has a larger load influence interval and stress sensitivity.
(4) The apparent void ratio has a good linear regression relationship with the conventional void ratio, while the apparent void ratio has a good exponential growth relationship with the compressibility. Pores with an average area of 2.5–4.5 μm^2^ have a more dominant effect on the physical and mechanical properties of soil than pores with an average area of 0.15–0.35 μm^2^.

## Figures and Tables

**Table 1:** Experimental schemes of the consolidation compression and SEM scanning of the samples.

Sample Types			Undisturbed Samples		Vertical Plane of Compressed Samples			
SEM Samples		Buried Depth (m)	Observation Direction		Consolidation Loads (kPa)			
			Vertical	Horizontal	100	400	800	1600
Number	DK-9-10 DK-23-24 DK-36-37 DK-44-45 DK-60-61	74.2–74.3 177.4–177.6 233.4–233.6 270.4–270.6 352.4–352.6	PV-1 PV-2 PV-3 PV-4 PV-5	PH-1 PH-2 PH-3 PH-4 PH-5	CV1-1 CV2-1 CV3-1 CV4-1 CV5-1	CV1-2 CV2-2 CV3-2 CV4-2 CV5-2	CV1-3 CV2-3 CV3-3 CV4-3 CV5-3	— CV2-4 CV3-4 CV4-4 CV5-4

**Table 2:** Conventional physical and mechanical parameters.

Number	Natural Density (kN/m^3^)	Porosity (%)	Compressibility (Mpa^−1^)	Permeability Coefficient (cm/s)	Preconsolidation Pressure (kPa)	Cohesion (kPa)	Internal Friction Angle (°)
DK-9-10	20.6	36.3	0.15	6.08 × 10^−7^	91	48.8	26.9
DK-23-24	21.3	34.1	0.18	3.66 × 10^−7^	196	51.6	24.6
DK-36-37	20.8	36.1	0.17	2.12 × 10^−8^	258	94.8	20.4
DK-44-45	21.3	34.5	0.16	1.17 × 10^−7^	272	89.7	23.5
DK-60-61	20.9	37.5	0.11	7.78 × 10^−7^	357	157.2	15.7

**Table 3:** Pore distribution under different consolidation loads (2000-times magnification).

Number	Consolidation Load/kPa	Total Number of Pores/1	SEM is Magnified 2000 Times The Content Distribution of Average Pore Area (%)				
			<1 μm^2^	1 μm^2^–2 μm^2^	2 μm^2^–4 μm^2^	4 μm^2^–8 μm^2^	>8 μm^2^
DK-9-10	0 100 400 800	1957 1228 1222 991	37.66 39.50 35.02 36.33	36.54 38.19 39.93 37.54	15.59 12.79 12.77 13.42	5.37 4.80 5.48 6.86	4.85 4.72 6.79 5.85
DK-23-24	0 100 400 800 1600	2186 2156 1514 1366 1526	38.20 37.94 37.91 38.09 38.79	38.24 36.13 34.54 35.26 34.86	15.60 15.82 15.46 16.76 15.73	4.53 5.66 6.61 5.59 6.49	3.43 4.45 5.48 4.30 4.13
DK-36-37	0 100 400 800 1600	1649 1337 1495 1441 1535	36.99 37.85 39.13 39.21 37.52	34.69 37.40 33.44 36.57 36.16	17.10 14.06 14.11 15.61 14.59	5.70 5.53 6.82 4.86 6.91	5.52 5.16 6.49 3.75 4.82
DK-44-45	0 100 400 800 1600	1731 2404 1494 1064 685	36.05 36.77 39.09 39.00 41.61	37.67 37.69 36.48 34.87 35.47	14.44 16.31 13.59 15.13 13.28	6.12 5.74 6.43 6.86 4.82	5.72 3.49 4.42 4.14 4.82
DK-60-61	0 100 400 800 1600	2481 2482 1469 1464 1093	36.88 41.34 41.59 41.53 39.52	35.83 39.40 39.48 39.75 42.63	15.80 14.18 12.66 12.70 13.72	6.73 3.30 3.68 4.44 3.39	4.76 1.77 2.59 1.57 0.73

**Table 4:** Pore distribution under different consolidation loads (10,000-times magnification).

Number	Consolidation Load/kPa	Total Number of Pores/1	SEM is Magnified 10,000 Times The Content Distribution of Average Pore Area (%)				
			<0.04 μm^2^	0.04 μm^2^–0.08 μm^2^	0.08 μm^2^–0.16 μm^2^	0.16 μm^2^–0.32 μm^2^	>0.32 μm^2^
DK-9-10	0 100 400 800	1064 657 501 285	35.24 30.14 31.74 33.33	35.43 37.44 36.93 33.33	15.13 15.22 16.57 14.04	6.95 8.68 7.98 8.42	7.24 8.52 6.79 10.88
DK-23-24	0 100 400 800 1600	1206 967 877 718 645	35.16 33.92 33.98 37.47 31.47	36.48 32.26 33.98 34.26 31.47	15.59 15.51 14.60 14.07 17.52	5.72 8.17 8.67 6.69 9.46	7.05 10.13 8.78 7.52 10.08
DK-36-37	0 100 400 800 1600	795 330 615 851 359	34.59 32.12 32.20 34.55 33.98	32.96 27.58 30.08 32.78 30.92	17.36 15.76 14.15 16.80 18.38	6.54 10.61 10.24 8.81 6.69	8.55 13.94 13.33 7.05 10.03
DK-44-45	0 100 400 800 1600	1027 1404 604 401 554	34.18 34.62 31.29 35.91 33.03	33.69 34.05 30.79 35.41 38.45	17.23 15.46 19.37 13.97 13.36	6.33 7.91 7.78 5.49 6.86	8.57 7.98 10.76 9.23 8.30
DK-60-61	0 100 400 800 1600	643 1121 607 903 577	31.70 33.36 35.09 34.33 38.30	30.43 34.52 30.81 30.01 37.09	19.15 16.59 16.47 17.05 13.17	7.02 7.94 8.57 10.74 5.55	11.70 7.58 9.06 7.86 5.89

## Data Availability

The data presented in this study are available on request from the corresponding author.

## References

[1] Mohammady M., Pourghasemi H.R., Amiri M. (2019). Land subsidence susceptibility assessment using random forest machine learning algorithm. Environ. Earth Sci..

[2] Yu H., Gong H., Chen B., Liu K., Gao M. (2020). Analysis of the influence of groundwater on land subsidence in Beijing based on the geographical weighted regression (GWR) model. Sci. Total Environ..

[3] Xue Y.Q., Zhang Y., Ye S.J., Li Q.F. (2003). Land Subsidence in China and its Problems. Quat. Sci..

[4] Poland J.F. (1984). Guidebook to Studies of Land Subsidence Due to Ground-Water Withdrawal.

[5] Galloway D.L., Jones D.R., Ingebritsen S.E. (1999). Land Subsidence in the United States.

[6] Rahmati O., Golkarian A., Biggs T., Keesstra S., Mohammadi F., Daliakopoulos I. (2019). Land subsidence hazard modeling: Machine learning to identify predictors and the role of human activities. J. Environ. Manag..

[7] Tolman C.F., Poland J.F. (1940). Ground–water. 1940, salt–water infiltration, and ground–surface recession in Santa Clara Valley, Santa Clara County, California. Eos Trans. Am. Geophys. Union.

[8] Zhang Y. (2002). One-Dimensional Model for Land Subsidence and its Solution. J. Eng. Geol..

[9] Liu Y. (2013). Spatiotemporal Evolution of Land Subsidence and Mechanism Discussion in the Yellow River Delta, China. Ph.D. Thesis.

[10] Zhang Y., Gong H., Gu Z., Wang R., Li X., Zhao W. (2014). Characterization of land subsidence induced by groundwater withdrawals in the plain of Beijing city. 2014, China. Hydrogeol. J..

[11] Gu K., Shi B., Liu C., Jiang H., Li T., Wu J. (2018). Investigation of land subsidence with the combination of distributed fiber optic sensing techniques and microstructure analysis of soils. Eng. Geol..

[12] Jiang H.T., Gu K., Yin J.H., Shi B., Ma J. (2018). Evaluation of Land Subsidence Based on Distributed Monitoring and SEM Analysis. Contemporary Issues in Geoenvironmental Engineering, Proceedings of the 1st GeoMEast International Congress and Exhibition, Sharm El Sheikh, Egypt, 15–19 July 2017.

[13] Fener M., Yesiller N. (2013). Vertical pore structure profile of a compacted clayey soil. Eng. Geol..

[14] Wang J.D., Li P., Ma Y., Vanapalli S.K. (2018). Evolution of pore-size distribution of intact loess and remolded loess due to consolidation. J. Soils Sediments.

[15] Zhao D., Gao Q.F., Hattab M., Hicher P.Y., Yin Z.Y. (2020). Microstructural evolution of remolded clay related to creep. Transp. Geotech..

[16] Gaboreau S., Robinet J.C., Prêt D. (2016). Optimization of pore-network characterization of a compacted clay material by TEM and FIB/SEM imaging. Microporous Mesoporous Mater..

[17] Tang C.-S., Lin L., Cheng Q., Zhu C., Wang D.-W., Lin Z.-Y., Shi B. (2020). Quantification and characterizing of soil microstructure features by image processing technique. Comput. Geotech..

[18] Di Remigio G., Rocchi I., Zania V. (2021). Scanning Electron Microscopy and clay geomaterials: From sample preparation to fabric orientation quantification. Appl. Clay Sci..

[19] Wei Y.-N., Fan W., Yu B., Deng L.S., Wei T. (2020). Characterization and evolution of three-dimensional microstructure of Malan loess. CATENA.

[20] Wang L., Li X.-A., Li L.-C., Hong B., Yao W., Lei H.-N., Zhang C. (2020). Characterization of the collapsible mechanisms of Malan loess on the Chinese Loess Plateau and their effects on eroded loess landforms. Hum. Ecol. Risk Assess. Int. J..

[21] Li X., Li L., Song Y., Hong B., Wang L., Sun J. (2019). Characterization of the mechanisms underlying loess collapsibility for land-creation project in Shaanxi Province. 2019, China—A study from a micro perspective. Eng. Geol..

[22] Lei H.Y., Xu Y.G., Jiang M.J., Jiang Y. (2020). Deformation and fabric of soft marine clay at various cyclic load stages. Ocean Eng..

[23] Dai C.-X., Zhang Q.-F., He S.-H., Zhang A., Shan H.-F., Xia T.D. (2021). Variation in Micro-Pores during Dynamic Consolidation and Compression of Soft Marine Soil. J. Mar. Sci. Eng..

[24] Zhang Y., Pu S., Li R.Y.M., Zhang J. (2020). Microscopic and mechanical properties of undisturbed and remoulded red clay from Guiyang. 2020, China. Sci. Rep..

[25] Wang Q., Chen J., Liu J., Yu M., Geng W., Wang P., Wu Z. (2020). Relationships between shear strength parameters and microstructure of alkaline-contaminated red clay. Environ. Sci. Pollut. Res..

[26] Wang Y., Yang H., Jing X. (2021). Structural Characteristics of Natural Loess in Northwest China and its Effect on Shear Behavior. Geotech. Geol. Eng..

[27] Ng C.W.W., Akinniyi D.B., Zhou C. (2020). Influence of structure on the compression and shear behaviour of a saturated lateritic clay. Acta Geotech..

[28] Chen H., Jiang Y., Niu C., Leng G., Tian G. (2019). Dynamic characteristics of saturated loess under different confining pressures: A microscopic analysis. Bull. Eng. Geol. Environ..

[29] Jiang Y., Chen H., Leng G. (2020). Dynamic elastic modulus of Xianyang loess based on microscopic analysis: A qualitative evaluation. Eur. J. Environ. Civ. Eng..

[30] Li L., Li X.-A., Wang L., Hong B., Shi J., Sun J. (2020). The effects of soil shrinkage during centrifuge tests on SWCC and soil microstructure measurements. Bull. Eng. Geol. Environ..

[31] Yan C.L., Zhang L., Tang Y.Q. (2020). Microscopic Experimental Analysis of the Accumulated Plastic Strain on a Silty Soil Around a Tunnel Under a Subway Loading. Geotech. Geol. Eng..

[32] Li Z., Liu Z., Cheng P., Li Z. (2021). Micro-characteristics and meso-shear mechanism of the soils of a slip zone in a landslide. Mech. Adv. Mater. Struct..

[33] Wang D., Yang C., Cheng G., Ma W., Zhang L., Petriaev A., Konon A. (2020). Experimental Study on Pore Water Pressure and Microstructures of Silty Clay Under Freeze-Thaw Cycles. Transportation Soil Engineering in Cold Regions.

[34] Zhang Y., Wang Z., Xue Y., Wu J. (2015). Visco-elasto-plastic compaction of aquitards due to groundwater withdrawal in Shanghai. 2015, China. Environ. Earth Sci..

[35] Shear D.L., Olsen H.W., Nelson K.R. (1992). Effects of Desiccation on the Hydraulic Conductivity Versus Void Ratio Relationship for a Natural Clay. Advances in Geotechnical Engineering, Transportation Research Record No. 1369.

[36] Hu R.L. (1995). Quantitative Microstructure Models of Clayey Soils and Their Engineering Behaviors.

[37] Zhou H. (2013). Study on Soft Soil Microstructure and Mechanism of Seepage and Consolidation in Pearl River Delta (PRD). Ph.D. Thesis.

[38] Liu C., Xu Q., Shi B., Gu Y.F. (2018). Digital image recognition method of rock particle and pore system and its application. Chin. J. Geotech. Eng..

[39] Tang C.S., Shi B., Wang B.J. (2008). Factors affecting analysis of soil microstructure using SEM. Chin. J. Geotech. Eng..

[40] Wang B.J., Shi B., Liu Z.B., Cai Y. (2004). Fractal study on microstructure of clayey soil by GIS. Chin. J. Geotech. Eng..

